# Willingness and influencing factors of herpes zoster vaccination intention among Chinese residents: a study based on framing effects

**DOI:** 10.3389/fpubh.2026.1849221

**Published:** 2026-07-01

**Authors:** Xiaohui Yang, Yuehui Li, Jianwei Liu, Shuaiqin Huang, Lingzhi Gui, Dehua Hu

**Affiliations:** 1 Nursing Department, The Third Xiangya Hospital of Central South University, Changsha, Hunan, China; 2 Department of Biomedical Information, School of Life Sciences, Central South University, Changsha, Hunan, China; 3 Department of Medical Parasitology, Xiangya School of Basic Medical Sciences, Central South University, Changsha, Hunan, China; 4 Xiangya School of Pharmaceutical Sciences, Central South University, Changsha, Hunan, China

**Keywords:** framing effect, herpes zoster, herpes zoster vaccine, vaccine acceptance intention, vaccine Price acceptance

## Abstract

**Objective:**

This study investigated how framing effects influence Chinese residents’ willingness to receive the herpes zoster vaccine.

**Methods:**

Based on gain-loss framing theory, two versions of the questionnaire were developed: a gain-framed version (Questionnaire A) and a loss-framed version (Questionnaire B). Using convenience sampling, 1,184 participants were enrolled through online and offline channels. Statistical analyses included independent-samples t-tests, one-way analysis of variance (ANOVA), multivariate regression, propensity score matching (PSM), and partial least squares structural equation modeling (PLS-SEM).

**Results:**

(1) Multivariate regression models showed high explanatory power, with adjusted *R*^2^ values of 0.796 (gain-framed), 0.822 (loss-framed), and 0.800 (overall). These values should be interpreted with caution because attitude is conceptually close to vaccination intention. (2) Two chain mediation pathways showed significant indirect associations: framing → information credibility → attitude → vaccination intention (FR → IC → AT→IT) and framing → vaccine price acceptance → attitude → vaccination intention (FR → PC → AT→IT) (both *p* < 0.001), suggesting that framing was associated with attitude and intention through respondents’ perceived message credibility and price acceptance. (3) A significant framing × gender interaction was observed (*B* = 0.446, *p* < 0.001). Stratified regression showed that loss-framed messages were associated with higher vaccination intention among males (*β* = 0.341; *p* < 0.001), whereas no statistically significant framing effect was found among females (*p* = 0.108). (4) A significant framing × herpes zoster (HZ) awareness interaction was also detected (*B* = 0.313; *p* = 0.002), indicating that individuals who were aware of HZ responded more positively to loss-framed messages. (5) Monthly income (*β* = 0.645; *p* < 0.001) and HZ awareness (*β* = 0.110; *p* < 0.001) were identified as important independent predictors.

**Conclusion:**

Framing effects on herpes zoster vaccination intention differed by gender and disease awareness. Gain-framed messages showed greater explanatory value in the full sample; however, loss-framed messages were associated with stronger vaccination intention among males and HZ-aware individuals, whereas no statistically significant framing effect was observed among females. The chain mediation pathways indicated statistically significant indirect associations among cognition, attitude, and behavioral intention, but did not confirm a psychological mechanism. Monthly income was identified as an important predictor of vaccination intention. These findings may provide a reference for developing more differentiated and targeted vaccine communication strategies.

## 1 Introduction

Herpes zoster is an infectious disease caused by reactivation of the herpes zoster virus and is commonly characterized by a painful rash (1). The global incidence of herpes zoster in the general population is about 3.5 per 1,000 person-years. In comparison, estimates for the Asia-Pacific region range from 3 to 10 per 1,000 person-years, with an annual increase of 2.5 to 5.0%. Worldwide, the hospitalization rate for herpes zoster ranges from 2 to 25 per 100,000 person-years, the mortality rate ranges from 0.017 to 0.465 per 100,000 person-years, and the recurrence rate ranges from 1 to 10% (2, 3). Among Chinese adults aged 50 years and above, the incidence of herpes zoster has been reported as 6.64 per 1,000 person-years (4).

Several studies have shown that the herpes zoster vaccine is highly effective and can provide long-term protection for older adults, patients with diverse underlying conditions, and individuals with prior herpes zoster recurrences (5–7). The coverage and effectiveness of the herpes zoster vaccine are important for reducing the healthcare burden, decreasing the risk of recurrence, and lowering the likelihood of related complications (8).

However, relatively few studies have examined Chinese residents’ willingness to receive the herpes zoster vaccine. Most existing studies were conducted within 6 months of vaccine approval, and studies published in 2021 and 2022 reported that residents’ willingness to receive vaccination increased markedly compared with the pre-approval period (9, 10). In addition, research has shown that herpes zoster causes greater harm than other skin infections, including higher pain intensity, complication rates, longer disease duration, and a higher recurrence risk. Although access to the vaccine has improved substantially, public willingness to receive the herpes zoster vaccine still has considerable room for improvement, and the main factors influencing vaccination decisions remain unclear.

Immunization programs in many countries have gradually shifted from the traditional child-centered “childhood immunization” model to a broader “life-course immunization” model that includes all population groups, emphasizing that vaccination should not be restricted to a single life stage but should be incorporated across the whole life course (11). The herpes zoster vaccine is an important component of preventive health services for older adults. Exploring willingness to receive this vaccine may provide useful practical evidence relevant to “healthy aging” and “life-course immunization” strategies, particularly in relation to aging, retirement, and preventive care for older adults (12).

Framing is a theory-based persuasive communication approach that aims to influence individuals’ cognition, judgment, attitudes, and behaviors by presenting logically equivalent information in different ways (13). It describes health behaviors from two perspectives: the benefits gained from taking action (i.e., gain-framed messages) and the losses or costs resulting from not taking action (i.e., loss-framed messages) (14). In essence, framing affects audiences’ emotions, attitudes, and decision-making processes through positive or negative, loss- or gain-oriented, and concrete or abstract expressions (15). In behavioral science research, framing effects are frequently used to influence individuals’ decision-making processes and behavioral tendencies (16, 17).

Research has shown that in prevention-oriented domains (e.g., sunscreen use for skin cancer prevention and smoking cessation), gain-framed messages are generally more effective than loss-framed messages (18, 19). As a preventive behavior, vaccination is considered to follow a similar pattern, in which gain-framed messages are more persuasive and exert a stronger influence on vaccination intention (20). In contrast, in disease-detection contexts, loss-framed messages usually have greater persuasive effects (21). Framing theory has been extensively applied in related areas, including influenza vaccination (22) and pneumococcal vaccination (23, 24). Moreover, several studies have examined public attitudes toward vaccination from a framing theory perspective (25).

Although framing theory has been applied in vaccination research, systematic studies specifically focusing on the herpes zoster vaccine remain limited, particularly in terms of large-scale empirical evidence. In addition, few studies have examined interactions effects between framing and sociodemographic characteristics (e.g., gender, education level, disease awareness), and the psychological processes underlying framing effects have not been fully clarified. Therefore, based on framing theory and the elaboration likelihood model (ELM), this study used an experimental survey design to systematically examine Chinese residents’ willingness to receive the herpes zoster vaccine and its influencing factors, including framing type, sociodemographic characteristics, information credibility, vaccine price acceptance, and social norms, to provide an empirical basis for improving vaccine communication strategies and enhancing vaccination intention.

## 2 Materials and methods

### 2.1 Questionnaire design

The questionnaire used in this study was designed to examine the public’s willingness to receive the herpes zoster vaccine and its influencing factors from the perspective of framing effects. The questionnaire first introduced the study’s purpose, presented information about herpes zoster through images and text, and described the current availability of herpes zoster vaccines on the market. The questionnaire included three main parts. The first part collected basic participant information, including gender, age, income, occupation, residence, education level, history of chronic diseases, and knowledge of herpes zoster disease and the herpes zoster vaccine. In the second part, participants were required to read four pieces of information developed according to the characteristics of the gain-loss framework, after which they reported their vaccination intention. In the third part, participants read further information on the price, effectiveness, and duration of protection of the herpes zoster vaccine, and then responded to items related to their vaccination intention.

### 2.2 Information design

All the information included in this study was developed based on a feature framework constructed through literature review, resident interviews, and expert consultations (26). The gain framework mainly emphasizes the benefits of taking action, whereas the loss framework highlights the costs of not taking action (27). Finally, three types of information stimuli were presented, as shown in Table 1. In addition, to control for the effect of information volume, the difference in word count between the two framing conditions was maintained within about 250 words. Detailed information is provided in the table.

**Table 1:** Stimulus information used in the questionnaire.

Item	Gain framing	Loss framing
Message 1	Getting vaccinated against herpes zoster can reduce the risk of infection. Even if one does get infected, the treatment duration is shorter, and the financial burden on the family is also lighter.	Without herpes zoster vaccination, the risk of infection is higher. Should infection occur, treatment takes longer, and the financial burden on the family is also greater.
Message 2	Getting vaccinated against herpes zoster can reduce the occurrence of complications such as rash, neuralgia, eye inflammation, and meningitis.	Without receiving the herpes zoster vaccine, the risk of developing complications such as rash, neuralgia, eye inflammation, and meningitis is higher.
Message 3	Getting vaccinated against herpes zoster can effectively prevent disease recurrence.	Without receiving the herpes zoster vaccine, the disease may recur, and symptoms may be more severe.

### 2.3 Variable measurement

This study examined the influence of framing (FR), attitudes (AT), intentions (IT), social norms (SN), and vaccine price acceptance (PC) on individual behavior and measured information credibility (IC). All constructs were evaluated using a five-point Likert scale, with higher scores indicating a stronger framing influence, a more positive attitude toward vaccination, stronger vaccination intention, greater perceived influence of social norms, and higher perceived information credibility. Measurement of all variables was based on previous research and slightly modified for the specific health behavior examined in this study (28, 29). The measurement of framing was adapted from Gantiva et al. (30); the measurement of social norms was modified from the study by Mir et al. (29); the measurement of information credibility was drawn mainly from Appelman and Sundar (31) and Sundar (32), and was assessed through the accuracy, authenticity, and reliability of information. The measurement of attitudes toward adopting health behaviors was also based on Mir et al. (29), and the intention to adopt health behaviors was adapted from Steffen et al. (33).

Regarding vaccine prices, this study referred to the prices of herpes zoster vaccines currently available on the market: the domestically produced live attenuated herpes zoster vaccine costs 1,600 ¥ per dose, while the imported recombinant herpes zoster vaccine costs 3,200 ¥ for the full two-dose series. Participants’ acceptance of herpes zoster vaccine prices was measured using a three-item Price Acceptance (PC) scale adapted from Wang et al. (34). The scale assessed willingness to receive vaccination under three price scenarios: (1) at the current market price, (2) at half the current price, and (3) when the vaccine was provided free of charge. The detailed items and their corresponding constructs are listed in Table 2.

**Table 2:** Factors and observed variables.

Factors	Code	Observed variables	References
Loss-Framing	L-FR1	I think failure to receive vaccination will increase my risk of getting herpes zoster.	Carlos et al. (30)
	L-FR2	I think failure to receive vaccination is not conducive to controlling herpes zoster symptoms.	
	L-FR3	I think failure to receive vaccination cannot effectively prevent the recurrence of herpes zoster.	
Gain-Framing	G-FR1	I think receiving a vaccination will reduce my risk of getting herpes zoster.	
	G-FR2	I think receiving a vaccination is conducive to controlling herpes zoster symptoms.	
	G-FR3	I think receiving a vaccination can effectively prevent the recurrence of herpes zoster.	
SN	SN1	My family influenced my decision to get vaccinated against herpes zoster.	Mir et al. (29)
	SN2	My friends and coworkers influenced my decision to receive the herpes zoster vaccine.	
	SN3	My physician’s recommendation influenced my decision to receive the herpes zoster vaccine.	
	SN4	Herpes zoster vaccination policies influenced my decision to receive the vaccine.	
IC	IC1	I think the above information is accurate.	Appelman and Sundar et al. (31, 32)
	IC2	I think the above information is true.	
	IC3	I think the above information is reliable.	
AT	AT1	In my opinion, vaccination is an effective measure to prevent herpes zoster transmission.	Mir et al. (29)
	AT2	I am willing to get vaccinated against herpes zoster.	
	AT3	I think everyone should get vaccinated against herpes zoster.	
PC	PC3	If the herpes zoster vaccine maintains its current price, I would be willing to receive the vaccination.	Wang et al. (34)
	PC4	If the price of the herpes zoster vaccine were halved, I would be willing to receive it.	
	PC5	If the herpes zoster vaccine is free, I would be willing to receive the vaccination.	
IT	IT1	Based on the above information, I have decided to get vaccinated against herpes zoster.	Steffen et al. (33)
	IT2	I would recommend my family and friends to get vaccinated against herpes zoster.	
	IT3	If recommended by doctors or the government, I will strictly follow the measures to get vaccinated against herpes zoster.	
	IT4	I intend to get vaccinated against herpes zoster to prevent the transmission of the virus.	

### 2.4 Structural equation modeling construction

**Figure 1:**
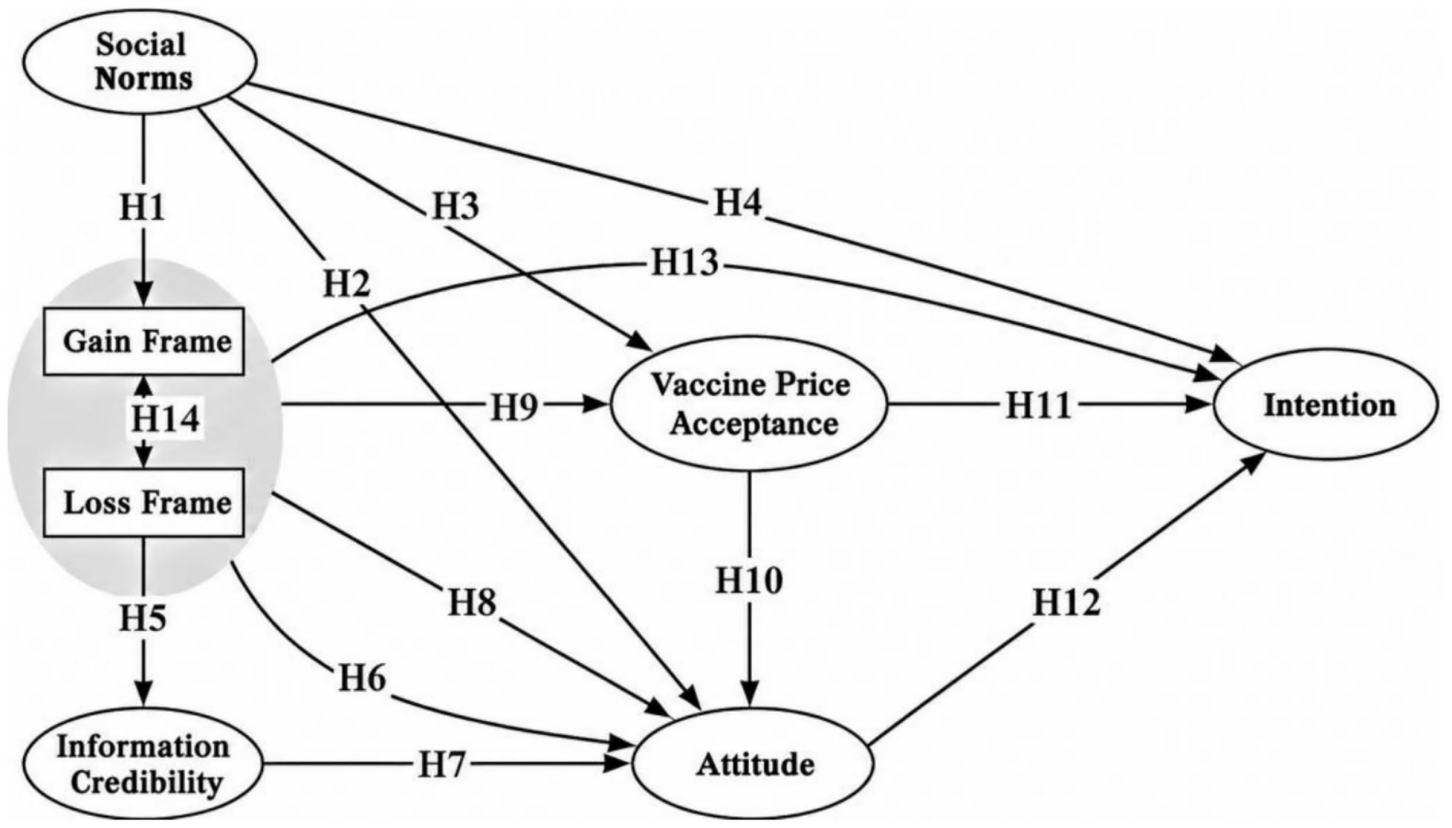
Structural equation model of this study.

To comprehensively analyze the associations between framing effects and the public’s willingness to receive the herpes zoster vaccine, a theoretical model was developed based on the external regulatory influence of social norms (SN) and the mediating roles of information credibility (IC) and vaccine price acceptance (PC) (Figure 1). The model comprises four categories of variables: framing (FR) as the independent variable, vaccination attitude (AT) and vaccination intention (IT) as the dependent variables, and IC and PC as mediating variables. SN was additionally incorporated into the model as an external influencing factor. This study further examined the relationships among these variables and proposed corresponding hypotheses.

SN refers to the rules or standards that guide individual behavior and help maintain social harmony by shaping responses to others’ expectations. Both social values and individual choices influence it. IC refers to an individual’s evaluation of the authenticity and credibility of information, which influences judgment and may mediate behavioral decision-making. When individuals receive information, they assess its credibility through different cognitive approaches. In framing research, price, attitude, and intention are commonly examined by designing corresponding framing conditions according to specific research objectives, among which the gain-loss framework is the most widely used form of health behavior framing (15). Framing influences individuals’ attitudes toward specific behaviors, which may in turn affect their behavioral intentions.

Vaccine price acceptance and willingness to receive vaccination under different pricing scenarios are important determinants of vaccination intention (35–37). Rothman argued that the loss framework (i.e., emphasizing the potential negative consequences of inaction) is more persuasive in the context of disease-detection behaviors. In contrast, the gain framework (i.e., emphasizing the potential positive outcomes of taking action) is more persuasive in prevention-oriented contexts. This perspective has generated extensive discussion in academic research and encouraged further investigation by many scholars. Lee further supported this theory through six well-designed experiments. These studies suggested that gain-framed information tends to be more persuasive when promoting specific behaviors, whereas loss-framed information is more effective when aiming to prevent a particular phenomenon. Herpes zoster vaccination is an effective preventive measure that can substantially reduce the risk of developing herpes zoster. Based on these findings, this study proposes hypotheses related to social norms, information credibility, framing, price acceptance, attitude, and intention, as presented in Table 3.

**Table 3:** Model path hypotheses.

Hypothesis	Hypothetical content
H1	Social norms influence the participants’ perception of the framing.
H2	Social norms influenced the participants’ attitudes toward herpes zoster vaccination.
H3	Social norms influence participants’ perceptions of the acceptability of the price of the herpes zoster vaccine.
H4	Social norms will influence the participants’ willingness to receive the herpes zoster vaccine.
H5	The framing affects participants’ judgment of the information’s credibility.
H6	Information credibility can mediate between framing and zoster vaccination attitudes.
H7	Information credibility can positively inlfuence attitudes toward herpes zoster vaccination.
H8	The framing influenced the participants’ attitudes toward herpes zoster vaccination.
H9	The framing influenced participants’ acceptance of the herpes zoster vaccine price.
H10	Participants’ acceptance of the herpes zoster vaccine price positively affected their attitude toward vaccination.
H11	Participants’ acceptance of the herpes zoster vaccine price positively influenced their willingness to vaccinate.
H12	Participants’ attitudes toward herpes zoster vaccination positively influenced their willingness to vaccinate.
H13	Participants’ price acceptance of the herpes zoster vaccine mediated the relationship between framing and vaccination intention.
H14	Gain framing is more persuasive in promoting vaccination behavior than loss framing.

### 2.5 Questionnaire distribution and collection

This study was approved by the Institutional Review Board of the School of Life Sciences, Central South University (Approval No. 2023–1-52). It was conducted in strict accordance with the Declaration of Helsinki. A convenience sampling method was used to recruit participants. The research team enrolled voluntary participants through online channels (e.g., WeChat, QQ, and online survey platforms such as WJX) and offline channels (e.g., paper-based questionnaires distributed in retirement communities).

Two versions of the questionnaire were administered: a gain-framed questionnaire (Questionnaire A) and a loss-framed questionnaire (Questionnaire B). Participants were assigned to either Questionnaire A or Questionnaire B with equal probability (1:1). For online participants, assignment was automatically completed via the survey platform’s built-in randomization function. For offline participants, the assignment was conducted by randomly selecting one questionnaire from a pre-shuffled stack of paper questionnaires, with Questionnaire A and Questionnaire B mixed at a 1:1 ratio. All participants were unaware of the questionnaire version they would receive before completing the survey. To minimize cross-contamination, participants were informed that those who had completed one version of the questionnaire should not complete the other.

The inclusion criteria were as follows: (1) participants who provided signed informed consent and voluntarily agreed to participate in the survey, and (2) participants who were able to complete the questionnaire independently. The exclusion criteria were as follows: (1) incomplete questionnaire responses; (2) clear logical inconsistencies in responses; and (3) repeated responses or a completion time of less than 1 min.

Before the formal survey, a pilot study involving 60 participants was conducted to confirm that respondents could read the framing instructions without difficulty, understand the questionnaire content, and complete the survey independently. Most participants reported that the framing information was easy to understand and that the questionnaire was the appropriate length. A total of 1,300 questionnaires were distributed, of which 1,184 valid questionnaires were ultimately retained. A total of 116 questionnaires were excluded for the following reasons: (1) incomplete questionnaire responses: 88 questionnaires; (2) clear logical inconsistencies in responses: 23 questionnaires; and (3) repeated responses or a completion time of less than 1 min: 5 questionnaires.

### 2.6 Statistical analysis

SPSS 27.0 and SmartPLS 4.0 were used for data analysis. To evaluate the validity of the measurement model, factor loadings of the observed variables and the average variance extracted (AVE) of the latent variables were used to assess convergent validity. Two-tailed tests were performed to examine the significance of path coefficients and moderating effects.

## 3 Results

### 3.1 Variable measurement

SmartPLS 4.0 was used to perform the structural equation modeling analysis. The results indicated that the measurement model was reliable. All Cronbach’s *α* values exceeded 0.7 (minimum = 0.778, maximum = 0.900), and all composite reliability (CR) values exceeded 0.8 (minimum = 0.871, maximum = 0.938), indicating adequate internal consistency and composite reliability of the measurement model.

With respect to convergent validity, the factor loadings of all observed variables exceeded 0.7 (minimum = 0.702, maximum = 0.933), and the average variance extracted (AVE) for each construct was greater than 0.5 (minimum = 0.634, maximum = 0.834), indicating acceptable convergent validity of the measurement model (Table 4).

**Table 4:** Psychometric properties of the measurement models.

Variables	Items	Loadings	Gain			Loss			
			CR	AVE	Cronbach’s alpha	Loadings	CR	AVE	Cronbach’s alpha
AT	AT1	0.919	0.938	0.834	0.9	0.928	0.935	0.827	0.894
	AT2	0.928				0.933			
	AT3	0.892				0.864			
IC	IC1	0.903	0.891	0.732	0.816	0.908	0.871	0.693	0.778
	IC2	0.791				0.746			
	IC3	0.869				0.835			
FR	IF1	0.919	0.931	0.818	0.889	0.912	0.936	0.831	0.898
	FR2	0.898				0.908			
	FR3	0.896				0.914			
IT	IT1	0.922	0.922	0.748	0.885	0.89	0.914	0.73	0.873
	IT2	0.875				0.893			
	IT3	0.721				0.702			
	IT4	0.925				0.914			
PC	PC3	0.885	0.908	0.767	0.847	0.829	0.896	0.742	0.824
	PC4	0.922				0.925			
	PC5	0.817				0.826			
	SN1	0.838	0.874	0.634	0.809	0.815	0.916	0.733	0.878
	SN2	0.793				0.879			
SN	SN3	0.749				0.868			
	SN4	0.801				0.861			

All factor loadings were significant at *p* < 0.001. CR, composite reliability; AVE, average variance extracted.

To evaluate discriminant validity, the heterotrait-monotrait (HTMT) ratio of correlations was examined (38). Following recent methodological recommendations, bias-corrected confidence intervals were calculated based on 10,000 bootstrap subsamples (39, 40). As shown in Table 5, the upper bounds of the 97.5% bias-corrected confidence intervals for the HTMT were below the conservative threshold of 0.90 for most construct pairs.

**Table 5:** HTMT bias-corrected confidence intervals.

Construct pair	HTMT	97.5% CI (bias-corrected)
FRAME ↔ AT	0.127	[0.094, 0.171]
FRAME ↔ IC	0.176	[0.124, 0.235]
FRAME ↔ IT	0.036	[0.015, 0.053]
FRAME ↔ PC	0.086	[0.041, 0.131]
FRAME ↔ SN	0.143	[0.106, 0.181]
IC ↔ AT	0.865	[0.824, 0.900]
IC ↔ IT	0.792	[0.745, 0.834]
IC ↔ PC	0.639	[0.578, 0.692]
IC ↔ SN	0.464	[0.403, 0.522]
IT ↔ AT	0.943	[0.921, 0.963]
IT ↔ PC	0.868	[0.833, 0.899]
IT ↔ SN	0.639	[0.584, 0.693]
PC ↔ AT	0.769	[0.729, 0.807]
PC ↔ SN	0.583	[0.525, 0.639]
SN ↔ AT	0.577	[0.520, 0.631]

FRAME is a single-item experimental manipulation variable (0 = gain-framed, 1 = loss-framed). Bias-corrected confidence intervals were calculated using 10,000 bootstrap subsamples. The 97.5% upper bound for AT ↔ IT was 0.963, which slightly exceeded the conservative threshold of 0.90 (39). This finding reflects the theoretically expected strong association between attitude and intention within the TPB framework (41).

Regarding the relationship between attitude (AT) and intention (IT), the HTMT upper bound was 0.963, slightly exceeding the 0.90 threshold. This finding is consistent with theoretical expectations because, within the Theory of Planned Behavior (TPB), attitude is conceptually and empirically one of the strongest predictors of intention, and previous meta-analyses have reported correlations typically ranging from 0.60 to 0.80 between these constructs (41).

Methodological researchers have noted that the 0.90 threshold is conservative and may be relaxed when constructs are theoretically closely related (42). Therefore, the elevated HTMT value between AT and IT likely reflects their theoretical proximity rather than a lack of measurement validity.

All other construct pairs demonstrated HTMT upper bounds below 0.90 (ranging from 0.171 to 0.900), supporting satisfactory discriminant validity.

### 3.2 Sample characteristics and balance test of random grouping

A total of 1,184 participants were included in this study, comprising 537 males (45.4%) and 647 females (54.6%). The age distribution was mainly concentrated in the 40–49 years (31.8%) and 50–59 years (30.7%) age groups, whereas participants aged ≤40 years accounted for 24.0% and those aged ≥60 years accounted for 13.5%. Regarding educational attainment, 302 respondents (25.5%) had completed middle school education or below, 265 (22.4%) had completed high school education, 223 (18.8%) had an associate college education, 285 (24.1%) held a bachelor’s degree, and 109 (9.2%) held a master’s degree or above. In terms of monthly income, the largest subgroup consisted of participants earning 5,001–10,000 ¥ per month (473 participants, 39.9%).

Regarding herpes zoster awareness, 72.7% of participants were aware of the disease, 58.1% knew about the herpes zoster vaccine, and 17.7% were aware of its price. Regarding perceived affordability (measured separately from the Price Acceptance scale), 395 participants (33.4%) considered the price reasonable, 644 (54.4%) considered it relatively expensive, and 145 (12.2%) considered it very expensive. For the imported recombinant herpes zoster vaccine, 25 participants (2.1%) considered the price reasonable, 554 (47.0%) considered it relatively expensive, and 605 (51.1%) considered it very expensive. Overall, participants perceived the domestic vaccine as substantially more affordable than the imported one.

To assess the effectiveness of random assignment, the distributions of key sociodemographic characteristics and herpes zoster-related awareness variables were compared between the gain-framed and loss-framed groups. Categorical variables were analyzed using the chi-square test, with *α* = 0.05. The results revealed significant differences between the two groups in terms of gender (*χ*^2^ = 13.61, *p* < 0.001), age group (*χ*^2^ = 47.06, *p* = 0.033), marital status (*χ*^2^ = 26.82, *p* < 0.001), educational level (*χ*^2^ = 12.84, *p* = 0.012), occupation (*χ*^2^ = 10.34, *p* = 0.035), monthly income (*χ*^2^ = 23.79, *p* < 0.001), herpes zoster awareness (*χ*^2^ = 44.22, *p* < 0.001), and herpes zoster vaccine price awareness (*χ*^2^ = 8.48, *p* = 0.004), indicating that random assignment did not fully achieve baseline balance between the groups. Given these baseline imbalances, comparisons between the framing groups should be interpreted as relying primarily on statistical adjustment rather than on a fully balanced randomized comparison. Therefore, subsequent analyses employed multivariate regression and propensity score matching (PSM) to adjust for observed covariate differences and reduce potential confounding in the study findings. Nevertheless, it should be acknowledged that statistical adjustment cannot fully replace a rigorously equivalent control-group design, and the validity of the framing-effect comparison remains dependent on the assumption of no unmeasured confounding. Detailed results are presented in Tables 6, panels A, B.

**Table 6:** Sample characteristics and balance test between the two groups.

Variables	Total sample (*n* = 1,184)	Gain (*n* = 590)	Loss (*n* = 594)	*χ* ^2^	*p*	Cramer’s V	Is it balanced?
Panel A							
Gender				13.61	<0.001	0.107	NO
Male	537 (45.4%)	236 (40.0%)	301 (50.7%)				
Female	647 (54.6%)	354 (60.0%)	293 (49.3%)				
Age group				47.06	<0.001	0.199	NO
≤40	284 (24.0%)	132 (22.4%)	152 (25.6%)				
40 ~ 49	376 (31.8%)	146 (24.7%)	230 (38.7%)				
50 ~ 59	364 (30.7%)	202 (34.2%)	162 (27.3%)				
≥60岁	160 (13.5%)	110 (18.6%)	50 (8.4%)				
Marital status				26.82	<0.001	0.151	NO
Unmarried	171 (14.4%)	103 (17.5%)	68 (11.4%)				
Married	955 (80.7%)	446 (75.6%)	509 (85.7%)				
Divorce	29 (2.4%)	16 (2.7%)	13 (2.2%)				
Widowed	29 (2.4%)	25 (4.2%)	4 (0.7%)				
Educational background				12.84	0.012	0.104	NO
Middle school and below	302 (25.5%)	171 (29.0%)	131 (22.1%)				
High school	265 (22.4%)	129 (21.9%)	136 (22.9%)				
Associate college	223 (18.8%)	113 (19.2%)	110 (18.5%)				
Bachelor’s degree	285 (24.1%)	120 (20.3%)	165 (27.8%)				
Master’s degree and above	109 (9.2%)	57 (9.7%)	52 (8.8%)				
Occupation				10.34	0.035	0.093	NO
Farmers	159 (13.4%)	83 (14.1%)	76 (12.8%)				
Self-employed	313 (26.4%)	164 (27.8%)	149 (25.1%)				
Enterprises/institutions	409 (34.5%)	201 (34.1%)	208 (35.0%)				
Retirement	124 (10.5%)	46 (7.8%)	78 (13.1%)				
Others	179 (15.1%)	96 (16.3%)	83 (14.0%)				
Monthly income (¥)				23.79	<0.001	0.142	NO
≤2000	231 (19.5%)	112 (19.0%)	119 (20.0%)				
2001 ~ 5,000	253 (21.4%)	116 (19.7%)	137 (23.1%)				
5,001 ~ 8,000	473 (39.9%)	273 (46.3%)	200 (33.7%)				
>8,000	227 (19.2%)	89 (15.1%)	138 (23.2%)				
Panel B							
Whether to learn about the herpes zoster				44.22	<0.001	0.193	NO
Yes	861(72.7%)	480(81.4%)	381 (64.1%)				
NO	323(27.3%)	110(18.6%)	213 (35.9%)				
Whether infected with the herpes zoster				0.02	0.897	0.004	Yes
Yes	199(16.8%)	100(16.9%)	99 (16.7%)				
NO	985(83.2%)	490(83.1%)	495 (83.3%)				
Whether to learn about the herpes zoster vaccine				0.75	0.386	0.025	Yes
Yes	719(60.7%)	351(59.5%)	368 (62.0%)				
NO	465(39.3%)	239(40.5%)	226 (38.0%)				
Whether to learn about the price of the herpes zoster vaccine				8.48	0.004	0.085	NO
Yes	254(21.5%)	106(18.0%)	148 (24.9%)				
NO	930(78.5%)	484(82.0%)	446 (75.1%)				
History of chronic disease				0.39	0.534	0.018	Yes
Yes	125(10.6%)	59(10.0%)	66 (11.1%)				
NO	1,059(89.4%)	531(90.0%)	528 (88.9%)				
Recruitment method				0.38	0.537	0.018	Yes
Online	771(60%)	379(64.2%)	392 (66.0%)				
Offline	413(40%)	211(35.8%)	202 (34.0%)				

Categorical variables are expressed as *n* (%). *p*-values were derived from the chi-square test. Cramer’s V indicates the strength of association. “Balanced” was defined as *p* > 0.05.

### 3.3 Main effect and moderating effect of framing on vaccination intention

#### 3.3.1 Single-factor analysis: differences in vaccination intention across subgroups

A total of 1,184 valid questionnaires were obtained, among which 590 used gain-framed messages, and 594 used loss-framed messages. Independent-samples t-tests were used to compare variables between two groups, whereas one-way ANOVA was applied for variables with more than two groups. The univariate analysis (Table 7, panels A, B) showed that, under loss-framed messages, males reported significantly greater vaccination intention than females (4.465 vs. 3.185; *t* = 20.416, *p* < 0.01); under gain-framed messages, females reported significantly higher vaccination intention than males (3.924 vs. 3.656, *t* = −3.71, *p* < 0.01). Education level and monthly income were both significantly and positively associated with vaccination intention (both *p* < 0.01). Age, marital status, and a history of chronic disease did not significantly affect vaccination intention. Participants with previous herpes zoster infection or greater knowledge of the disease were more likely to report willingness to receive vaccination. Awareness of the herpes zoster vaccine was also positively associated with vaccination intention.

**Table 7:** Effect of demographic characteristics on herpes zoster vaccination intention.

Variables		Gain				Loss			
		Total	*M*(SD)	*F*(*T*)	*p*	Total	*M*(SD)	*F*(*T*)	*p*
Panel A									
Gender									
Male		236	3.656 (0.89)	−3.71	<0.01	301	4.465 (0.636)	20.416	<0.01
Female		354	3.924 (0.818)			293	3.185 (0.871)		
Age group									
≤40		132	3.784 (0.899)	0.386	0.763	152	3.85 (1.007)	1.34	0.26
40–49		146	3.771 (0.85)			230	3.747 (1.047)		
50–59		202	3.853 (0.805)			162	3.949 (0.896)		
≥60		110	3.852 (0.913)			50	3.81 (0.996)		
Marital status									
Unmarried		103	3.782 (0.906)	1.115	0.342	68	4.007 (0.894)	1.024	0.38
Married		446	3.844 (0.84)			509	3.811 (1.007)		
Divorce		16	3.5 (0.785)			13	3.923 (0.703)		
Widowed		25	3.69 (0.99)			4	3.438 (1.712)		
Educational background									
Middle school or below		171	3.449 (0.984)	14.67	<0.01	131	2.985 (0.983)	79.535	<0.01
High school		129	3.777 (0.88)			136	3.465 (0.908)		
Associate college		113	4.064 (0.694)			110	4.096 (0.812)		
Bachelor’s degree		120	4.056 (0.68)			165	4.368 (0.616)		
Master’s degree and above		57	4.018 (0.605)			52	4.688 (0.423)		
Occupation									
Farmers		83	3.033 (0.871)	31.04	<0.01	76	2.688 (0.888)	100.65	<0.01
Self-employed		164	3.976 (0.842)			149	3.755 (0.916)		
Enterprises/institutions		201	4.056 (0.671)			208	4.442 (0.576)		
Retirement		46	4.109 (0.718)			78	4.266 (0.765)		
Others		96	3.583 (0.832)			83	3.093 (0.79)		
Panel B									
Monthly income (¥)									
≤2000	112	2.627 (0.658)	253.6	<0.01	119	2.656 (0.894)	346.65	<0.01	
2000–5,000	116	3.532 (0.634)			137	3.243 (0.607)			
5,001–10,000	273	4.265 (0.521)			200	4.308 (0.461)			
≥10,000	89	4.312 (0.48)			138	4.75 (0.429)			
Whether to learn about the herpes zoster virus									
Yes	480	3.897(0.809)	20.34	<0.01	381	4.299(0.707)	19.537	<0.01	
No	110	3.468(0.972)			213	3.001(0.884)			
Whether infected with the herpes zoster virus									
Yes	100	4.145 (0.686)	4.994	<0.01	99	4.654 (0.485)	14.917	<0.01	
No	490	3.75 (0.874)			495	3.67 (0.989)			
Whether to learn about the herpes zoster vaccine									
Yes	351	3.949(0.795)	4.513	<0.01	337	4.298(0.707)	16.978	<0.01	
No	239	3.622(0.908)			257	3.077(0.928)			
Whether to learn about the price of the herpes zoster vaccine									
Yes	106	3.995 (0.863)	2.374	<0.01	104	4.323 (0.727)	8.478	<0.01	
No	484	3.778 (0.852)			490	3.672 (1.018)			
History of chronic disease									
Yes	59	3.941 (0.706)	1.383	0.17	66	3.75 (1.078)	−0.726	0.47	
No	531	3.803 (0.872)			528	3.844 (0.984)			

#### 3.3.2 Multiple regression analysis: the predictive effect of framing on vaccination intention

Given the baseline imbalances between the gain- and loss-framed groups identified in the balance checks, multiple linear regression analyses were conducted as a supplementary robustness check to adjust for potential confounding variables when examining the predictive effect of framing on vaccination intention. These analyses complement the primary SEM approach by testing whether the observed relationships remained stable after controlling for demographic covariates. The results are shown in Table 8.

**Table 8:** Multiple linear regression analysis of vaccination intention by framing and covariates.

Variable	Gain-framed (*n* = 590)	Loss-framed (*n* = 594)	Full sample (*n* = 1,184)
B (β)	B (β)	B (β)	
Constant	0.987***	0.355***	0.725***
AT	0.367*** (0.426)	0.538*** (0.496)	0.468*** (0.486)
FR	0.331*** (0.394)	0.061* (0.060)	0.163*** (0.176)
Frame type (loss vs. gain)	—	—	−0.110*** (−0.059)
Gender (male vs. female)	−0.016 (−0.009)	0.141** (0.071)	−0.015 (−0.008)^1^
Education level	−0.050*** (−0.079)	0.010 (0.013)	−0.059*** (−0.084)^1^
Monthly income	0.154*** (0.173)	0.304*** (0.322)	0.240*** (0.261)
Age group	−0.003 (−0.003)	0.054** (0.049)	0.022 (0.024)
Herpes zoster virus knowledge (aware vs. unaware)	−0.035 (−0.016)	0.185*** (0.089)	0.088** (0.042)
Frame × Gender	—	—	0.220*** (0.084)
Frame × Education	—	—	0.097*** (0.096)
Model fit	*F*(7, 582) = 328.52***	*F*(7,586) = 391.40***	*F*(10, 1,173) = 474.56***
Adj. R^2^	0.796	0.822	0.8
D–W value	1.867	1.822	1.868

*p < 0.05, **p < 0.01, ***p < 0.001. B, unstandardized regression coefficient; β, standardized regression coefficient in parentheses. “—” indicates that the variable was not included in the model. ^1^Gender and education level were mean-centered before the interaction terms were constructed in the full-sample model. Frame type: loss-framed = 1, gain-framed = 0. All VIF values were less than 5, indicating no serious multicollinearity.

The models explained substantial variance in vaccination intention (adjusted *R*^2^ = 0.796 for the gain-framed group, 0.822 for the loss-framed group, and 0.800 for the full sample). Notably, these high adjusted *R*^2^ values should be interpreted with caution, as attitude is conceptually closely associated with vaccination intention, which may partly inflate the explained variance.

In the gain-framed group, attitude (AT) and framing (FR) significantly and positively predicted vaccination intention: AT (*B* = 0.367, *β* = 0.426, *p* < 0.001) and FR (*B* = 0.331, *β* = 0.394, *p* < 0.001). Monthly income (*B* = 0.154, *β* = 0.173, *p* < 0.001) was also a significant positive predictor, whereas education (*B* = −0.050, *β* = −0.079, *p* < 0.001) showed a significant negative association.

In the loss-framed group, AT (*B* = 0.538, *β* = 0.496, *p* < 0.001) and FR (*B* = 0.061, *β* = 0.060, *p* = 0.031) were also significant predictors of vaccination intention. In addition, gender (male, *B* = 0.141; *β* = 0.071; *p* = 0.005), monthly income (*B* = 0.304; *β* = 0.322; *p* < 0.001), age group (*B* = 0.054; *β* = 0.049; *p* = 0.005), and herpes zoster awareness (*B* = 0.185; *β* = 0.089; *p* < 0.001) were significant positive predictors.

In the full-sample interaction model, the main effect of framing type was significant (*B* = −0.110, *β* = −0.059, *p* < 0.001), indicating that the loss-framed group had significantly lower vaccination intention than the gain-framed group. Significant interaction effects were observed for Framing × gender (*B* = 0.220, *p* < 0.001) and Framing × Education (*B* = 0.097, *p* < 0.001), suggesting that the association between framing and vaccination intention differed by gender and education level.

Hypothesis H14 (gain framing is more persuasive than loss framing) was not supported. Although the main effect was significant (*B* = −0.110, *p* < 0.001), the effect size was small (*β* = −0.059), and significant interactions (Framing × gender, Framing × Education, and Framing × Herpes Zoster Awareness) were identified. Gender-stratified analysis showed that the framing effect was significant only among males (*β* = 0.341, *p* < 0.001) but not among females (*p* = 0.108), indicating that the advantage of gain framing was conditional rather than universal.

#### 3.3.3 Propensity score matching (PSM) robustness check

To adjust for observed covariate differences and reduce baseline imbalance, this study applied 1:1 nearest-neighbor PSM. Before matching, significant differences were observed between the two groups across several variables (mean standardized difference = 18.7%). After matching, the standardized differences for all covariates decreased to less than 10% (mean = 5.2%) (Table 9), indicating improved balance in the observed characteristics. The post-PSM regression results (Table 10) showed that the main effect of framing remained significant (*B* = −0.126, *p* = 0.039), and the Framing × gender interaction effect was also significant (*B* = 0.446, *p* < 0.001), supporting the robustness of the interaction.

**Table 9:** Comparison of balance test results between the gain group and loss group before and after PSM.

Variables	Before matching(*n* = 1,184)			After matching(*n* = 774)		
	*χ*^2^/*t*	*p*	Standardized difference (%)	*χ*^2^/*t*	*p*	Standardized difference (%)
Gender	13.61	<0.001	21.5	0.87	0.350	6.7
Age group	47.06	<0.001	31.4	0.92	0.820	3.4
Educational background	12.84	0.012	13.9	3.98	0.409	8.6
Occupation	10.34	0.035	4.9	2.32	0.677	2.1
Monthly income (¥)	23.79	<0.001	2.6	1.72	0.632	9.3
Herpes zoster virus awareness	44.22	<0.001	39.4	0.10	0.747	2.3
Whether to learn about the price of the herpes zoster vaccine	8.48	0.004	17.0	0.28	0.599	3.8
Mean	—	—	18.7	—	—	5.2

Standardized differences are expressed as absolute values (%). Values < 10% indicate an adequate balance. PSM reduced the mean difference from 18.7 to 5.2%.

**Table 10:** Multivariate linear regression analysis results after PSM (*n* = 774).

Variables	B	SE	*β*	*t*	*p*	95% CI
Intercept	2.151	0.146		14.779	<0.001	(1.865, 2.436)
Framing type (Loss = 1, Gain = 0)	−0.126	0.061	−0.070	−2.070	0.039	(−0.245, −0.006)
Male dummy variable (male01, Male = 1)	−0.177	0.060	−0.099	−2.949	0.003	(−0.295, −0.059)
Framing × Male	0.446	0.090	0.217	4.936	<0.001	(0.268, 0.623)
Age group	0.038	0.022	0.042	1.723	0.085	(−0.005, 0.081)
Education level	0.013	0.022	0.019	0.613	0.540	(−0.029, 0.056)
Monthly income (¥)	0.578	0.028	0.645	20.366	<0.001	(0.522, 0.634)
Occupation	−0.003	0.018	−0.005	−0.186	0.852	(−0.039, 0.032)
Herpes zoster virus awareness (Aware = 1)	0.221	0.052	0.110	4.278	<0.001	(0.119, 0.322)
Vaccine price perception	−0.024	0.052	−0.011	−0.454	0.650	(−0.127, 0.079)

Dependent variable: Vaccination intention. Framing type: Gain = 0, Loss = 1. Model fit: *F*(9, 764) = 118.96, *p* < 0.001, Adj. *R*^2^ = 0.579.

#### 3.3.4 Interaction effect tests and gender-stratified analysis

To determine whether the effect of framing on vaccination intention varied according to demographic and cognitive characteristics, interaction-term models were constructed using the PSM-matched sample. The results are presented in Table 11. The interaction effect tests revealed that the Framing × gender and Framing × Herpes Zoster Awareness interactions were statistically significant, indicating that males and individuals who were aware of herpes zoster were more strongly influenced by loss-framed messages. In contrast, the interactions between Framing and Education, and between Framing and Monthly Income, were not statistically significant.

**Table 11:** Interaction effect tests between framing type and demographic variables.

Interaction term	*B*	*SE*	*p*	95% CI	Interpretation
Framing × Gender (Male = 1)	0.446	0.090	<0.001	(0.268, 0.623)	Males are more influenced by loss framing
Framing × Education level	0.047	0.034	0.174	(−0.021, 0.114)	Education level moderation is not significant
Framing × Monthly income	0.034	0.046	0.465	(−0.057, 0.125)	Income moderation is not significant
Framing × Herpes zoster virus awareness (Aware = 1)	0.313	0.098	0.002	(0.120, 0.506)	Aware individuals are more influenced by loss framing

Each model included one interaction term and all the covariates. Framing: Gain = 0, Loss = 1.

To further clarify the significant Framing × gender interaction, gender-stratified regression analyses were conducted. The results are shown in Table 12. The stratified analysis demonstrated that the framing effect was significant among males (*β* = 0.341, *p* < 0.001), with higher vaccination intention observed under loss-framing than under gain-framing. In contrast, the framing effect was not significant among females (*β* = −0.105, *p* = 0.108). These findings are consistent with the interaction analysis and further suggest that the influence of framing on vaccination intention differs significantly by gender. Given the baseline imbalances between the two groups, these findings are based on statistical adjustments and should be interpreted with the study design limitations in mind.

**Table 12:** Regression analysis stratified by gender after PSM.

Variables	Level	*N*	Gain group	Loss group	*β*	*SE*	*p*	95%CI	*R* ^2^
Gender	Male	381	184	197	0.341***	0.063	<0.001	0.218 ~ 0.465	0.586
Gender	Female	393	203	190	−0.105	0.065	0.108	−0.232 ~ 0.023	0.533

β, effect of loss-framing (vs. gain-framing). ***p < 0.001. All models were adjusted for covariates.

### 3.4 Structural equation model

#### 3.4.1 Model fit and explained variance

**Figure 2:**
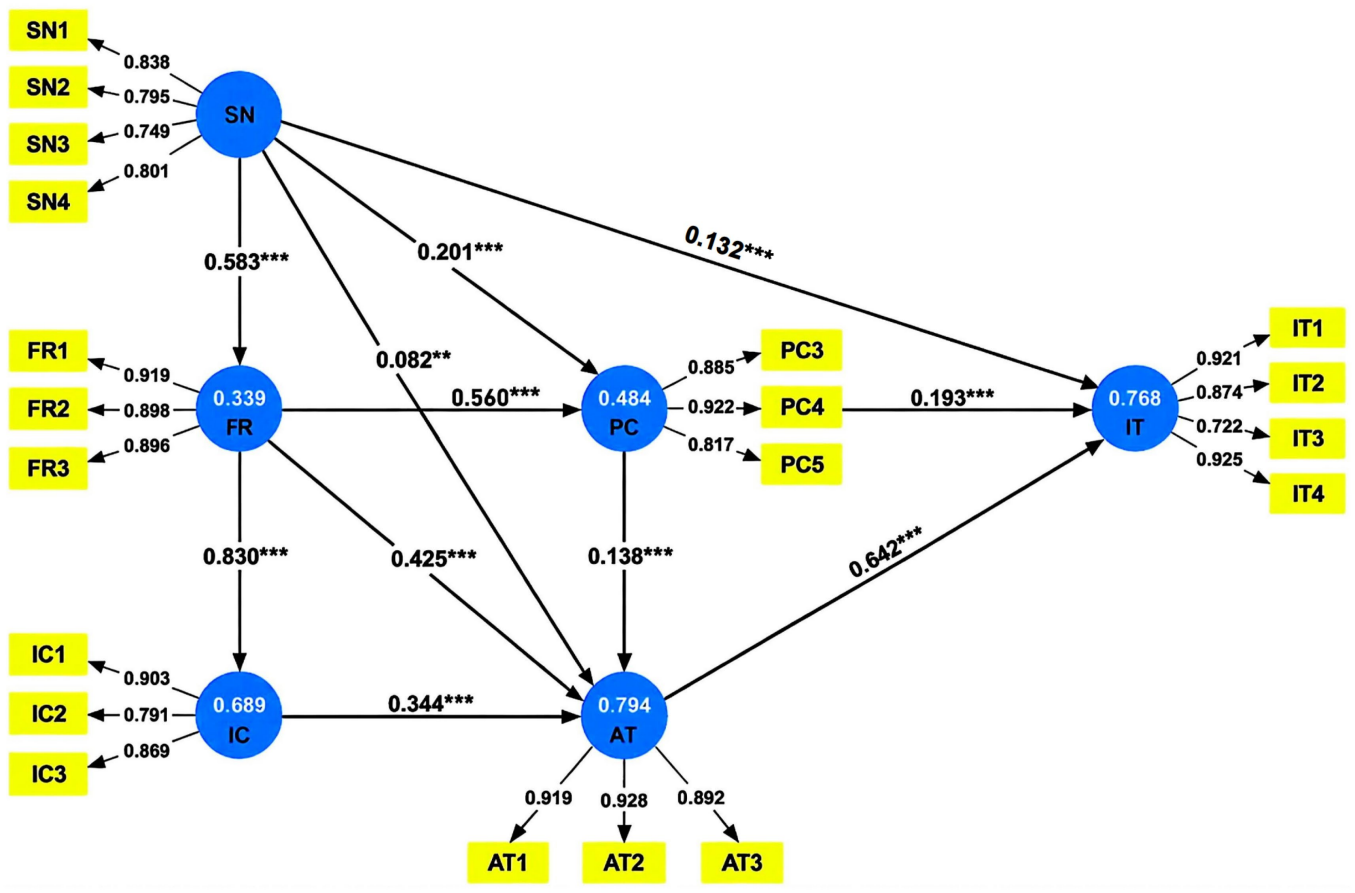
Research model for hypothesis testing under gain framing. *p < 0.05, **p < 0.01, ***p < 0.001.

**Figure 3:**
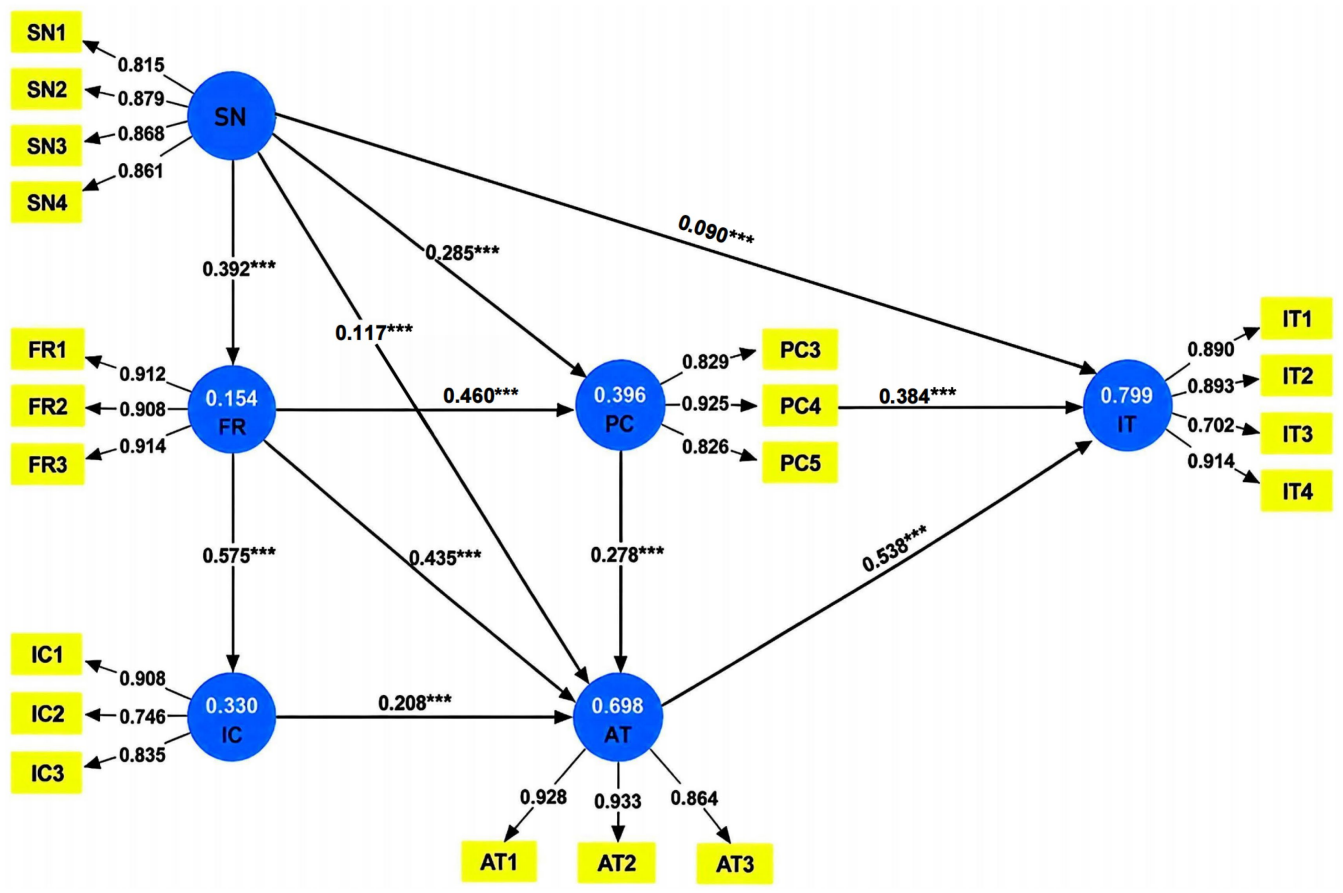
Research model for hypothesis testing under loss framing. *p < 0.05, **p < 0.01, ***p < 0.001.

In this study, partial least squares structural equation modeling (PLS-SEM) was used to examine the path relationships among the latent variables. Significance testing was conducted using bias-corrected and accelerated (BCa) bootstrapping with 5,000 resamples, and the significance level was set at *α* = 0.05 (two-tailed). The model converged after three iterations, and the path coefficients are presented in Figures 2, 3.

Explained variance (*R*^2^): The model demonstrated good explanatory power for the endogenous latent variables. Under the gain-framed condition, the explained variance (R^2^) for Framing (FR), Information Credibility (IC), Attitude (AT), Vaccine Price Acceptance (PC), and Intention to Vaccinate (IT) was 33.9, 68.9, 79.4, 48.4, and 76.8%, respectively. Under the loss-framed condition, the corresponding *R*^2^ values were 15.4, 33.0, 69.8, 39.6, and 79.9%, respectively. These results indicate strong explanatory power for attitude and vaccination intention (*R*^2^ > 0.50).

Predictive relevance (*Q*^2^): All *Q*^2^ values for the endogenous latent variables were greater than zero (Table 13), indicating satisfactory predictive relevance of the model.

**Table 13:** *Q*^2^ and *f*^2^ values of latent variables.

Frame	Variable	SSO	SSE	*Q* ^2^	Path	*f* ^2^
Gain	AT	1770	607.229	0.657	IC → AT	0.165
					FR → AT	0.239
					PC → AT	0.045
					SN → AT	0.020
	IC	1770	886.499	0.499	FR → IC	2.215
	FR	1770	1282.759	0.275	SN → FR	0.514
	IT	2,360	1016.107	0.569	AT→IT	0.786
					PC → IT	0.079
					SN → IT	0.047
	PC	1770	1121.818	0.366	FR → PC	0.401
					SN → PC	0.052
Loss	AT	1782	762.903	0.572	IC → AT	0.094
					FR → AT	0.330
					PC → AT	0.153
					SN → AT	0.034
	IC	1782	1381.467	0.225	FR → IC	0.494
	FR	1782	1556.829	0.126	SN → FR	0.181
	IT	2,376	1002.293	0.578	AT→IT	0.747
					PC → IT	0.386
					SN → IT	0.030
	PC	1782	1265.459	0.290	FR → PC	0.297
					SN → PC	0.114

SN (social norms) is the only exogenous variable; therefore, its *Q*^2^ value is not reported. *f*^2^ ≥ 0.02 = small, ≥ 0.15 = medium, ≥ 0.35 = large (Cohen, 1988).

Effect size (*f*^2^): According to Cohen’s criteria (*f*^2^ ≥ 0.02 = small, ≥ 0.15 = medium, ≥ 0.35 = large effect), the effect sizes for each path is summarized in Table 13.

Gain-framed condition: Framing exerted a large effect on Information Credibility (*f*^2^ = 2.215) and Vaccine Price Acceptance (*f*^2^ = 0.401), and a medium effect on Attitude (*f*^2^ = 0.239). Social Norms had a large effect on Framing (*f*^2^ = 0.514). Attitude exerted a large effect on Intention to Vaccinate (*f*^2^ = 0.786).

Loss-framed condition: Framing exerted a large effect on Information Credibility (*f*^2^ = 0.494) and Attitude (*f*^2^ = 0.330), and a medium effect on Vaccine Price Acceptance (*f*^2^ = 0.297). Social Norms had a medium effect on Framing (*f*^2^ = 0.181). Both Attitude (*f*^2^ = 0.747) and Vaccine Price Acceptance (*f*^2^ = 0.386) had strong effects on Intention to Vaccinate.

#### 3.4.2 Path coefficients and hypothesis testing

The standardized coefficients (*β*), *t* values, 95% confidence intervals, and hypothesis testing results for each path are shown in Table 14. All hypothesized paths met the following criteria: *p* < 0.01, 95% CI excluding zero, and *t* > 1.96. The main results are summarized as follows:

**Table 14:** Path coefficient and hypothesis testing analysis.

Hypothesis	Path	Frame	Path factor	*t*	95% Confidence interval	*p*
H1	SN → FR	Gain	0.583^***^	18.295	[0.517, 0.644]	***
		Loss	0.392^***^	10.542	[0.321, 0.464]	***
H2	SN → AT	Gain	0.082^**^	3.240	[0.034, 0.134]	**
		Loss	0.117^***^	3.937	[0.060, 0.176]	***
H3	SN → PC	Gain	0.201^***^	4.254	[0.109, 0.294]	***
		Loss	0.285^***^	8.101	[0.218, 0.354]	***
H4	SN → IT	Gain	0.132^***^	4.719	[0.079, 0.189]	***
		Loss	0.090^***^	3.739	[0.044, 0.138]	***
H5	FR → IC	Gain	0.830^***^	48.808	[0.794, 0.861]	***
		Loss	0.575^***^	14.778	[0.497, 0.650]	***
H7	IC → AT	Gain	0.344^***^	8.472	[0.262, 0.419]	***
		Loss	0.208^***^	6.413	[0.143, 0.270]	***
H8	FR → AT	Gain	0.425^***^	11.018	[0.350, 0.500]	***
		Loss	0.435^***^	10.959	[0.356, 0.511]	***
H9	FR → PC	Gain	0.560^***^	12.530	[0.470, 0.645]	***
		Loss	0.460^***^	12.445	[0.387, 0.531]	***
H10	PC → AT	Gain	0.138^***^	4.913	[0.081, 0.190]	***
		Loss	0.278^***^	8.283	[0.213, 0.343]	***
H11	PC → IT	Gain	0.193^***^	5.128	[0.120, 0.265]	***
		Loss	0.384^***^	11.984	[0.322, 0.447]	***
H12	AT → IT	Gain	0.642^***^	18.534	[0.570, 0.708]	***
		Loss	0.538^***^	15.769	[0.468, 0.601]	***

***p < 0.001, **p < 0.01. CI, confidence interval; SN, subjective norm; FR, framing; IC, information credibility; PC, vaccine price acceptability; AT, attitude; IT, vaccination intention. All hypotheses were supported at *p* < 0.05.

Hypothesis testing for Social Norms (SN) (H1–H4): The direct effects of SN on FR (*β* = 0.583/0.392), AT (*β* = 0.082/0.117), PC (*β* = 0.201/0.285), and IT (*β* = 0.132/0.090) were all statistically significant (all *p* < 0.01). Therefore, H1–H4 were supported.

Hypothesis testing for framing (FR) (H5, H8, H9): FR showed the strongest effect on IC (*β* = 0.830/0.575, *p* < 0.001). The direct effects of FR on AT (*β* = 0.425/0.435) and PC (*β* = 0.560/0.460) were also significant (all *p* < 0.001). Therefore, H5, H8, and H9 were supported.

Hypothesis testing for mediating variables (H7, H10, H11): The effect of IC on AT was stronger under the gain-framed condition (*β* = 0.344 vs. 0.208). The effects of PC on AT (*β* = 0.138 vs. 0.278) and IT (*β* = 0.193 vs. 0.384) were stronger under the loss-framed condition (all *p* < 0.001). Therefore, H7, H10, and H11 were supported.

Prediction of intention by attitude (H12): AT showed the strongest path to IT among all paths in the model (*β* = 0.642/0.538, *p* < 0.001). Therefore, H12 was supported.

#### 3.4.3 Mediation effect test

PLS-SEM was applied to examine the direct, indirect, and total effects of framing on vaccination intention (Table 15).

**Table 15:** Indirect, direct, and total effects.

Frame	Path	Direct effect	Indirect effect	Total effect	Hypothesis
Gain	FR → AT	0.425^***^	––	0.788***	––
	FR → IC → AT	––	0.286^***^	––	H6 Supported
	FR → PC → AT	––	0.077^***^	––	Supported
	FR → PC → IT	––	0.124^***^	0.676^***^	H13Supported
	FR → AT → IT	––	0.298^***^	––	Supported
	FR → PC → AT → IT	––	0.054^***^	––	Supported
	FR → IC → AT → IT	––	0.200^***^	––	Supported
Loss	FR → AT	0.435^***^	––	0.682^***^	––
	FR → IC → AT	––	0.119^***^	––	H6 Supported
	FR → PC → AT	––	0.128^***^	––	Supported
	FR → PC → IT	––	0.177^***^	0.544^***^	H13Supported
	FR → AT → IT	––	0.234^***^	––	Supported
	FR → PC → AT → IT	––	0.069^***^	––	Supported
	FR → IC → AT → IT	––	0.064^***^	––	Supported

***p < 0.001. FR, framing; IC, Information Credibility; PC, Vaccine Price Acceptability; AT, Attitude; IT, Vaccination Intention. Since there is no direct path from FR to IT in the model, the total effect of FR on IT (0.676 for Gain, 0.544 for Loss) equals the sum of all indirect effects from FR to IT.

Total effect: Under the gain-framed condition, the total effect of framing on attitude was *β* = 0.788 (*p* < 0.001), and its total effect on vaccination intention was *β* = 0.676 (*p* < 0.001). Under the loss-framed condition, the corresponding total effects were *β* = 0.682 and *β* = 0.544, respectively (both *p* < 0.001).

Hypothesis H6 (FR → IC → AT): The indirect effect of framing on attitude through information credibility was statistically significant in both the gain-framed group (*β* = 0.286, *p* < 0.001) and the loss-framed group (*β* = 0.119, *p* < 0.001). Therefore, H6 was supported.

Hypothesis H13 (FR → PC → IT): The indirect effect of framing on vaccination intention through vaccine price acceptance was statistically significant in both the gain-framed group (*β* = 0.124, *p* < 0.001) and the loss-framed group (*β* = 0.177, *p* < 0.001). Therefore, H13 was supported.

The serial mediation path (FR → IC → AT → IT) showed statistically significant indirect associations in both the gain-framed group (*β* = 0.200) and the loss-framed group (*β* = 0.064). The serial mediation path (FR → PC → AT → IT) was also statistically significant in both the gain-framed group (*β* = 0.054) and the loss-framed group (*β* = 0.069) (all *p* < 0.001).

### 3.5 Correlation analysis between vaccine price acceptance and vaccination intention

As shown in Table 16, vaccine price acceptance was positively and significantly correlated with vaccination intention across all price scenarios, including the current-price, half-price, and free-vaccine scenarios (all *p* < 0.01). Further linear regression analysis (Table 17) indicated that price acceptance had the strongest association with increased vaccination intention in the free-vaccine scenario, with the regression coefficient being slightly higher in the gain-framed group than in the loss-framed group (0.351 vs. 0.341, *p* < 0.001).

**Table 16:** Correlation analysis between vaccine price and vaccination intention.

Frame	Variables	PC3	PC4	PC5	IT
Gain	PC3	1			
	PC4	0.790^**^	1		
	PC5	0.528^**^	0.627^**^	1	
	IT	0.624^**^	0.624^**^	0.615^**^	1
Loss	PC3	1			
	PC4	0.711^**^	1		
	PC5	0.459^**^	0.659^**^	1	
	IT	0.616^**^	0.731^**^	0.673^**^	1

**p < 0.01 (two-tailed). PC3, at the current market price; PC4, at half the current price; PC5, when the vaccine is provided free of charge; IT = Intention.

**Table 17:** Linear regression analysis of vaccine price on vaccination intention.

Frame	Variables	*B*	*t*	*p*	Tolerance	VIF	*F*	Adj. R^2^	D–W
Gain	PC3	0.316	6.693	<0.001	0.375	2.668	202.94^***^	0.507	1.587
	PC4	0.154	2.995	0.003	0.315	3.170			
	PC5	0.351	9.439	<0.001	0.604	1.655			
Loss	PC3	0.202	5.587	<0.001	0.494	2.025	319.44^***^	0.617	1.951
	PC4	0.363	8.485	<0.001	0.353	2.829			
	PC5	0.341	10.078	<0.001	0.565	1.770			

***p < 0.001. B, unstandardized coefficient; VIF, variance inflation factor; D–W, Durbin–Watson statistic.

### 3.6 The influence of social norms on vaccination intention

The study showed that social norms, including family member influence (SN1), friends and colleagues (SN2), doctor recommendations (SN3), and government vaccination policies (SN4), were significantly associated with vaccination intention. As shown in Tables 18, 19, linear regression analysis indicated that under the gain frame, the influence of family members was stronger (*B* = 0.396 > *B* = 0.241, *p* < 0.001). In the loss frame, however, government vaccination policies showed a stronger association than family member influence (*B* = 0.291 > *B* = 0.214, *p* < 0.001), suggesting that the public may be more responsive to policy signals when potential health risks are emphasized. In the loss frame, doctor recommendations were also significantly associated with herpes zoster vaccination intention (*B* = 0.167, *p* = 0.002), suggesting that physician advice becomes more influential when potential losses are highlighted. The influence of friends and colleagues was comparatively weaker. These findings provide practical implications for developing more effective vaccination communication strategies.

**Table 18:** Analysis of the correlation between social norms and vaccination intention.

Framing	Variables	SN1	SN2	SN3	SN4	IT
Gain	SN1	1				
	SN2	0.603^**^	1			
	SN3	0.460^**^	0.494^**^	1		
	SN4	0.518^**^	0.487^**^	0.523^**^	1	
	IT	0.582^**^	0.437^**^	0.403^**^	0.506^**^	1
Loss	SN1	1				
	SN2	0.645^**^	1			
	SN3	0.588^**^	0.712^**^	1		
	SN4	0.565^**^	0.682^**^	0.670^**^	1	
	IT	0.443^**^	0.404^**^	0.451^**^	0.489^**^	1

**p < 0.01 (two-tailed). SN1, family member influence; SN2, friends and colleagues; SN3, doctor recommendations; SN4, government vaccination policies; IT, Intention.

**Table 19:** Linear regression analysis of social norms on vaccination intention.

Frame	Variables	*B*	*t*	*p*	Tolerance	VIF	*F*	Adj. *R*^2^	D–W
Gain	SN1	0.396	9.273	<0.001	0.561	1.781	98.08^***^	0.397	1.510
	SN2	0.045	1.066	0.287	0.562	1.778			
	SN3	0.073	1.818	0.070	0.641	1.561			
	SN4	0.241	5.898	<0.001	0.613	1.632			
Loss	SN1	0.214	4.520	<0.001	0.537	1.861	60.71^***^	0.287	2.010
	SN2	−0.051	−0.903	0.367	0.373	2.681			
	SN3	0.167	3.113	0.002	0.417	2.399			
	SN4	0.291	5.670	<0.001	0.456	2.194			

***p < 0.001. B, unstandardized coefficient; VIF, variance inflation factor; D–W, Durbin–Watson statistic.

## 4 Discussion

The World Health Organization (WHO) and various national immunization programs have gradually shifted from the traditional “childhood immunization” framework toward a broader life-course immunization perspective, emphasizing that vaccination should extend across the human lifespan (43, 44). Herpes zoster vaccination is particularly relevant at a critical juncture in the life course of older adults: immunosenescence is a key biological basis of herpes zoster, with incidence doubling every 5 to 10 years after age 50; systematic changes in health-related behaviors accompany retirement; chronic disease management increases contact with healthcare systems; and the growing emphasis on “healthy aging” has further increased attention to preventive services (45, 46). However, substantial room remains to improve the Chinese residents’ willingness to receive the herpes zoster vaccine, and the main factors shaping vaccination decisions remain unclear.

This study explored the association between message framing and willingness to receive the herpes zoster vaccine and constructed a structural equation model to examine the roles of information credibility and vaccine price acceptance in shaping vaccination willingness and attitudes. The main findings are as follows: (1) the effect of message framing on vaccination willingness differed significantly by gender, with loss-framed messages being associated with greater vaccination intention among males, whereas no statistically significant framing effect was observed among females; (2) attitude and information credibility showed statistically significant indirect associations with message framing and vaccination willingness; (3) vaccine price acceptance was an important factor related to vaccination willingness, and the findings suggest the potential value of reducing price barriers; and (4) social norms, including family member recommendations, physician advice, and government policies, were significantly associated with vaccination willingness.

### 4.1 Overall comparison and boundary conditions of message framing effects

This study revealed that the main effect of message framing was significant in the full-sample interaction model (*B* = −0.110, *p* < 0.001), indicating that vaccination intention in the loss-framed group was significantly lower than that in the gain-framed group. However, Hypothesis H14 (gain framing is more persuasive than loss framing for vaccination behavior) was only partly supported: although the main effect reached significance, the effect size was small (*β* = −0.059), and significant interaction effects (Frame × gender, Frame × Education, and Frame × Cognition) were identified.

These findings suggest that the key question should not be “which frame is generally better” but rather “which frame works better for which population.” Gain framing appeared to be slightly stronger than loss framing in the overall sample, but this advantage was not universal. It was moderated by factors such as gender, education level, and disease cognition.

Post-PSM regression analysis further supported the robustness of the main framing effect (*B* = −0.126, *p* = 0.039), and the significant Frame × gender interaction (*B* = 0.446, *p* < 0.001) indicated that gender differences in framing effects persisted after adjustment for observed covariate differences.

### 4.2 Gender differences in framing effects

Multiple regression (Table 8) revealed that the effect of framing on vaccination intention differed significantly by gender, as reflected by the Frame ×gender interaction (*B* = 0.446, *p* < 0.001). One-way analysis (Table 7) indicated that under the loss frame, males reported greater intention than females (*t* = 20.416), whereas under the gain frame, females reported greater intention than males (*t* = −3.71). Hierarchical regression (Table 3) revealed that the loss frame significantly increased intention among males (*β* = 0.341, *p* < 0.001), whereas no statistically significant framing effect was observed among females (*p* = 0.108). These results are consistent with the gender difference hypothesis of information processing theory (47), which suggests that males may be more sensitive to loss-related information. In contrast, females may respond more positively to gain-related information.

Direct evidence explaining the mechanism behind this gender difference remains limited. One possible explanation is that the cognitive pattern of health risk perception among males is more oriented toward potential losses. Because herpes zoster can cause severe pain and long-term complications (e.g., postherpetic neuralgia), which substantially affect patients’ quality of life (48–50), males may be more concerned about the consequences of not receiving vaccination. Another possible explanation is that females tend to show greater caution and self-protective awareness when facing health-related issues (47); however, the framing effect among females did not reach statistical significance in this study. This finding differs from those of some previous studies, which may be due to differences in sample characteristics or cultural context. Notably, these interpretations remain speculative and require direct validation in future studies.

Although the psychological mechanisms underlying gender differences require further examination, this study’s findings may provide some guidance for vaccine communication. Specifically, loss-related information (e.g., health risks of non-vaccination) could be appropriately highlighted for male populations. In contrast, gain-related information (e.g., health benefits of vaccination) could be appropriately emphasized for female populations.

### 4.3 Relationship between disease awareness and vaccination intention

This study showed that participants with a history of herpes zoster infection or higher levels of disease awareness had significantly stronger vaccination intentions than those without such experience or with lower levels of disease awareness. These results are consistent with the “experience-behavior consistency” theory, which suggests that personal experience of disease-related suffering can strengthen prevention motivation. From the perspective of immunosenescence, the incidence of herpes zoster doubles every 5 to 10 years after age 50 (2, 3), indicating that individual disease risk increases substantially with age. Individuals with a more in-depth understanding of herpes zoster are more likely to recognize the severe pain and long-term complications of the disease and, therefore, may be more willing to reduce disease risk through vaccination (51). These findings are consistent with the concept of “healthy aging”; by enhancing disease awareness, effective health interventions can be implemented at key life stages (e.g., around retirement), which is relevant to life-course immunization perspectives. Furthermore, awareness of the herpes zoster vaccine significantly increases people’s vaccination intention (52, 53). These findings suggest that public health authorities should strengthen education on herpes zoster-related disease among middle-aged and older adults to improve their awareness of vaccination.

### 4.4 Influence of sociodemographic factors

Education level: Participants with a bachelor’s degree or higher had stronger vaccination intentions than those with a high school education or lower. These findings suggest that a higher education level may help individuals better understand the potential risks of herpes zoster and the importance of vaccination (54, 55). Consistent with the life-course immunization perspective, education level may influence an individual’s ability to access and process health information around the time of retirement. However, the cross-sectional design of this study does not directly test such dynamic transitions.

Income level: Monthly income was significantly and positively associated with vaccination intention and was identified as the strongest predictor in this study. This may be because the cost of the herpes zoster vaccine is more acceptable to higher-income groups (52). These findings are consistent with the concept of “preventive care” discussed earlier, as financial barriers may restrict some older adults from accessing necessary preventive health services.

Age and marital status: Age was significant only under the loss-framed condition, whereas marital status had no significant effect under either framing condition. Although the incidence of herpes zoster increases with age (2, 3), age itself was not a strong predictor of vaccination intention. Low vaccination uptake may result from mobility difficulties or limited awareness of the need for adult vaccines (56), rather than from age itself. These findings suggest that vaccination promotion for older adults should focus on improving accessibility and increasing awareness, rather than relying on age alone as the basis for recommendations.

### 4.5 Role of vaccine price acceptance

Structural equation modeling confirmed that vaccine price acceptance mediates the association between framing and vaccination intention. Under both gain-framed and loss-framed conditions, vaccine price acceptance was positively associated with vaccination intention. The effect size analysis showed that, under the loss-framed condition, the effect size of vaccine price acceptance on vaccination intention increased substantially (*f*^2^ = 0.386).

Notably, when the vaccine was priced at zero, the influence of income differences on vaccination intention clearly decreased. These findings suggest that reducing price barriers may narrow socioeconomic disparities and increase overall vaccination intention (57, 58). These findings are consistent with the goals outlined in the introduction of “reducing the healthcare burden, lowering recurrence risk, and decreasing related complications” (9). However, because this study used hypothetical price scenarios rather than real-world policy interventions, these results should be interpreted as indicating potential value rather than direct evidence of policy effectiveness.

### 4.6 Influence of social norms

This study showed that social norms were significantly associated with attitudes toward herpes zoster vaccination, acceptance of vaccine price, and vaccination intention. Specifically:

Family members: Under the gain-framed condition, family members’ influence was more evident (*B* = 0.396, *p* < 0.001). From a life-course perspective, family support networks may become more important after retirement.

Government vaccination policy: Under the loss-framed condition, the influence of government policy was the strongest (*B* = 0.291, *p* < 0.001), suggesting that the public may show stronger risk-avoidance responses when potential health risks are emphasized. Physician recommendations: Physician recommendations were significantly associated with vaccination intention under the loss-framed condition (*B* = 0.167, *p* = 0.002), indicating that professional advice may be more persuasive in loss-framed contexts. This finding is consistent with the observation of “more frequent contact with the healthcare system” during chronic disease management (59, 60).

Friends and colleagues: They exerted relatively weaker influence under both framing conditions, yet remained important components of social networks. These findings suggest that social norms and external advice play important roles in guiding older adults’ vaccination decisions. Collaborative communication through multiple channels and agents may foster a social atmosphere conducive to vaccination.

### 4.7 Mediation mechanisms: theoretical significance of the chain mediation pathways

The two serial mediating pathways identified in this study are consistent with the dual-route processing perspective of the Elaboration Likelihood Model (ELM) (61). According to the ELM, persuasion may occur through the central route (rational analysis of message arguments) and the peripheral route (heuristic processing of external cues) (61). In this study, framing may function as a peripheral cue and may activate both processing processes: on the one hand, framing may shape attitudes through the peripheral route by influencing individuals’ perceived information credibility (IC); on the other hand, framing may trigger more systematic value considerations by affecting individuals’ evaluation of vaccine price acceptance (PC). These two pathways eventually converge on attitude (AT) and are statistically associated with vaccination intention (IT). A growing body of research indicates that in health communication contexts, information credibility and affective or economic factors may jointly shape individuals’ health decisions (62, 63). These findings suggest that the persuasive effect of framing may not operate through a single pathway but through parallel associations involving peripheral heuristics and systematic evaluation, thereby extending the theoretical explanation of framing effects in the vaccination domain.

### 4.8 Limitations

This study has the following limitations:

(1) Sample representativeness. Convenience sampling resulted in an overrepresentation of highly educated individuals (54.6%) and older adults (aged >60 years, 13.5%). Given that education level emerged as a significant predictor of vaccination intention in the analyses, caution is warranted when generalizing the conclusions to populations with lower educational attainment. Therefore, the findings should be generalized with care, and future research should test the proposed model in more diverse and representative samples.

(2) Randomization imbalance. Balance checks revealed significant baseline differences between the gain-framed and loss-framed groups across multiple key variables (e.g., gender, age, education, income, and herpes zoster awareness), indicating that randomization did not achieve equivalent groups. Consequently, framing effect comparisons relied primarily on statistical adjustments (e.g., PSM, multivariate regression) rather than a strictly equivalent control group design. Although PSM partially mitigated these imbalances, it reduced the sample size and cannot fully rule out bias from unobserved confounders. Therefore, conclusions regarding the differential persuasiveness of gain versus loss frames should be interpreted with caution. Future studies should employ stratified randomization or larger sample sizes to ensure baseline equivalence.

## Data Availability

The original contributions presented in the study are included in the article/supplementary material, further inquiries can be directed to the corresponding author.
